# The Sixpenny Doctor

**Published:** 1897-01-30

**Authors:** 


The Hospital

A Journal of

The Medical Sciences and Hospital Administration,

Vol. XXI.—No. 540.] [Jan. 30, 1897.

### The Sixpenny Doctor.

For a medical journal which aspires to represent the very diverse feelings and desires of its numerous subscribers, the "Dialogue" is a useful editorial device. Whatever question is discussed both sides see their own ideas put in print, everyone is satisfied that the editor understands him, and no one is a bit the worse. Wherefore, we congratulate the *British Medical Journal* on its Dialogues on Current Questions. The very gist and essence of such essays is that they are devoid of finality, and commit no one.

In regard, however, to such a subject as cheap practice, it seems desirable to do something more definite than simply airing what is to be said on each side and leaving people to take their choice. Some sort of editorial pronouncement may reasonably be expected, and a journal may fairly be asked whether it draws the line at the traditional half-crown or is willing to shake hands with that "disgrace to the profession," the sixpenny doctor. Now, for our own part, we have no hesitation in speaking plainly, and in saying that the line between what is permissible and what cannot be allowed is not to be drawn in terms of fee, and only vaguely touches the amount which each man thinks a proper reward for his services and skill.

How the money is earned, in what company it is earned, and by what means the patients are tempted to pay it, seem to us far more important than its mere amount. The question of pence or guineas is beside the mark. The very first point which shows the absurdity of attempting to ostracise the sixpenny doctor on commercial grounds is that a mass of men who are among the most respectable members of the profession, and, in fact, are the principal Government medical officers all over the country, namely, the members of the Poor Law medical service, notoriously work for less than sixpence a visit. Then if one turns to the other aspect of the case, the ethics of seeing patients for less than a remunerative fee—in other words, the ethics of doing low price practice, or absolutely free practice, for ulterior personal ends—do we not see the most honoured in our ranks every day seeing patients absolutely for nothing? Yet, although general practitioners speak ill enough of the hospitals at which this is done, only a few will be found to say that the men of high qualifications who accept hospital appointments which entail the doing of it are sinning against the ethics of their profession. The more one looks at the matter the more clear does it become that, whatever theoretical standard people may choose to set up, as a matter of fact the estimation in which a man is held by his professional brethren is not regulated by the price he charges for his services, and that there is no fixed limit of fee below which a man may not go without becoming a "disgrace to his profession." If there be such a limit at all the professional conscience is usually more inclined to fix it in the upward than in the downward direction. Instances are known in which the acceptance of an obviously outrageous fee has laid men under the disapprobation of their fellows, as men willing to take advantage of man's, or, worse still, of woman's, weakness; but as for the man who takes his sixpence, one is sorry he should have to do so, but if it is honestly accepted for honest work the profession cannot consistently protest.

What it can and does protest against is that work should be dishonestly done; that patients should be attracted by dishonest promises, should be allured by the arts of the advertiser, and that members of the profession should sell their services to others to be bartered by them in their own way for their own profit and advantage. These are the things that are really "infamous in a professional respect," whether they are done by consultants or by sixpenny doctors, and with these the question of fee has no concern.

The difficulty of the sixpence lies in the fact that to make it pay patients must be attracted in such multitude that the temptations to illegitimate practice are much increased. Advertising, the distribution of handbills, touting, and various disreputable methods of attracting "custom," undoubtedly prevail to a considerable extent among the sixpenny dispensaries. Moreover, the overmastering necessity of coming into touch with a large circle of the sixpenny-paying public brings into existence that abomination, the "branch dispensary," and that beggarly appendage of an overdriven profession, the unqualified assistant of the sixpenny doctor. So is medicine dragged in the mire, and we need not wonder that, seeing what it so often leads to, "cheap practice" stinks in the nostrils of so many members of our profession, and that the "sixpenny dispensary" is looked upon by so many as, in itself, abominable.

We must insist, however, that it is the methods and accompaniments which are wrong, and not the cheapness, and that if, in higher walks of practice, patients are drawn to West-end consulting-rooms by unworthy devices, are then made to think lightly of their own medical men, and are beguiled into paying heavy fees for unnecessary services, the size of the fee in no way lessens the dishonour.

No man should belittle his professional brother; no man should do anything to take from him his patient; no man should deal dishonestly, as he does by undertaking more work than he can conscientiously perform, or offering medicine for fees at which it cannot be provided of good quality. But as to the actual fee which he considers a fitting remuneration for the exercise of his professional skill—so that it is such as will enable him to exercise that skill to the best of his knowledge and ability—that is a matter which must depend on conditions personal to himself, and with that we do not see that we have any right to dogmatise or interfere.

No member of the medical profession has a right to offer short measure either of skill or physic to a public which puts trusts in his being a qualified man. It is no defence for so doing to say that he has done the best he can afford for sixpence. But if, when he does his best, he is content with the small reward which sixpence offers, that is his own concern.

